# Supplementary material for: De novo mutations mediate phenotypic switching in an opportunistic human lung pathogen
Source: Nat Commun. 2025 Jul 23;16:6799. doi: 10.1038/s41467-025-61168-4 (PMC12287342; doi:10.1038/s41467-025-61168-4)
Supplement: Supplementary file 1 — Supplementary Information [file 41467_2025_61168_MOESM1_ESM.pdf]

Supplementary Figures

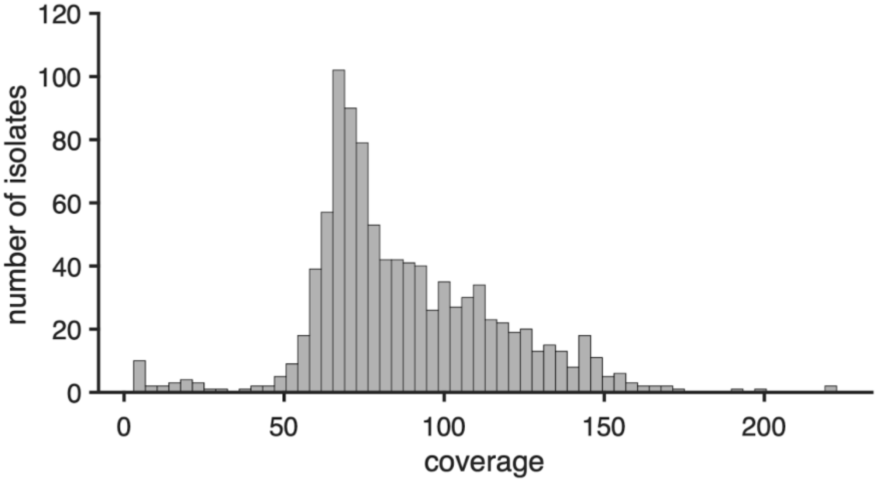

Supp. Figure 1: 987 *B. dolosa* isolates were sequenced at an average depth of 88x
A histogram catalogs the coverage of every isolate sequenced for this project. This dataset was sequenced to a mean depth of 87.7x with a standard deviation of 29.3x.

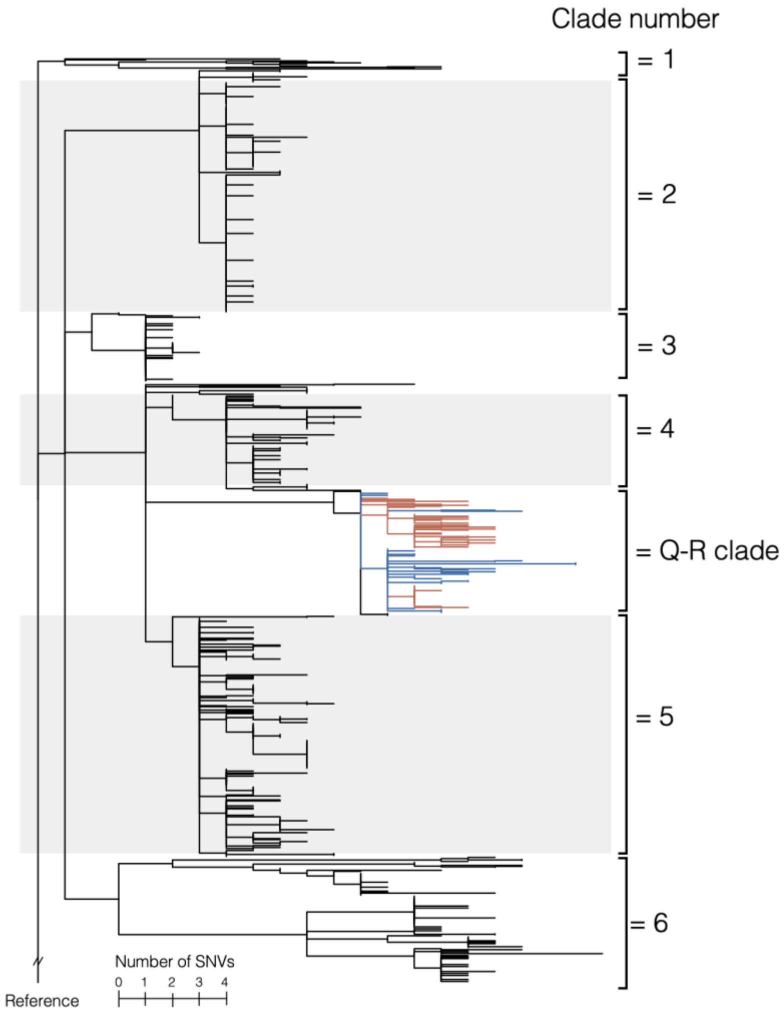

Supp. Figure 2: A SNV phylogeny of Patient J's, Q's, & R's *B. dolosa* isolates displays transmission patterns. More detailed version of the subclade of **Fig. 2a** shaded in gray. We created a maximum parsimony SNV phylogeny of all sequenced isolates from Patients J, Q, and R. Branch colors indicate patient, with Patient Q marked as blue, Patient R as red, and Patient J as black. Six major clades are observed within J's isolates. These are numbered for reference in other supplementary materials. Eleven samples share 3 SNVs common to clades 4, 5, and Q-R but lack shared SNVs that define each of these clades; these samples are therefore not included in any numbered clade.

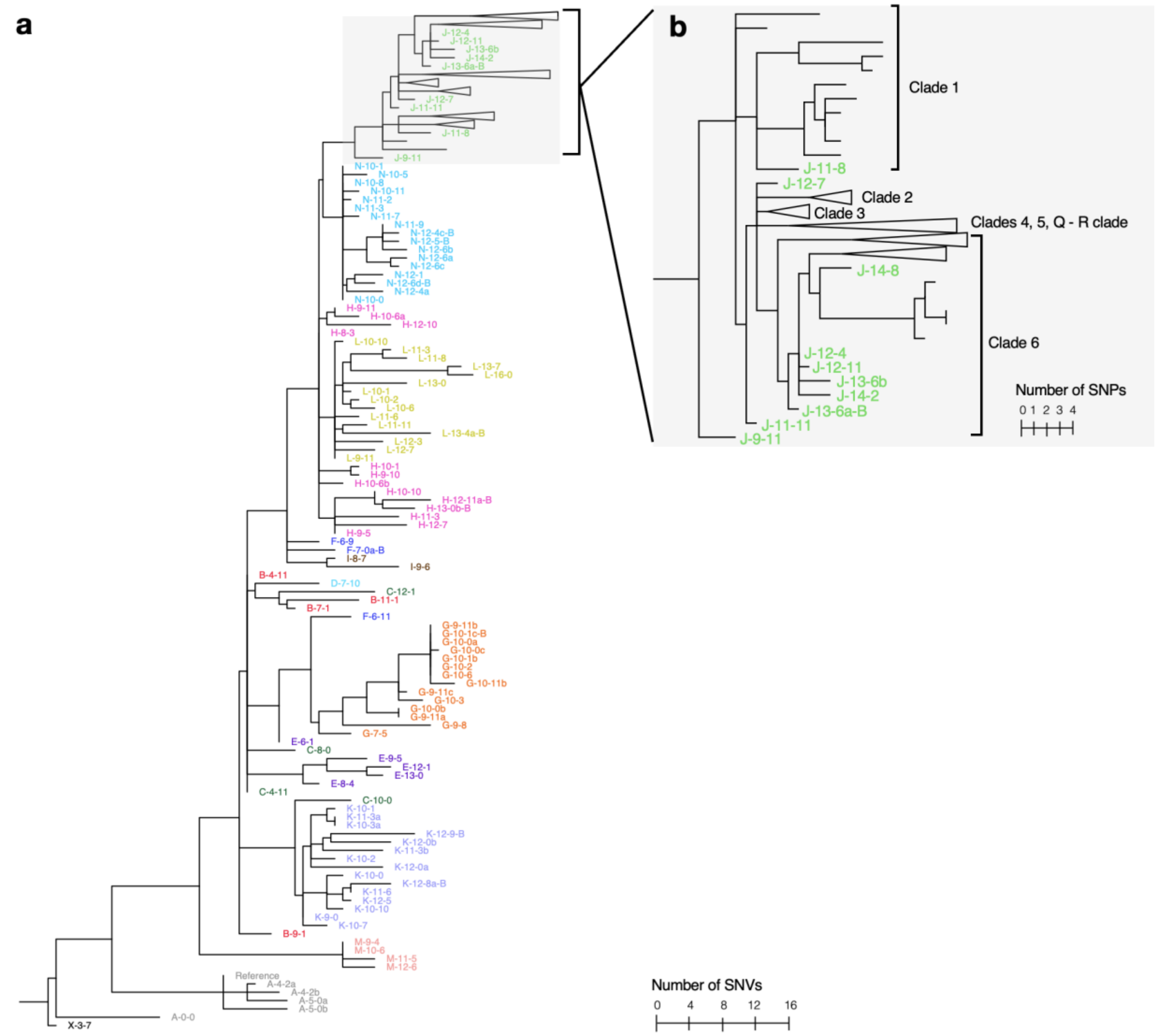

Supp. Figure 3: Comparing Patient J's *B. dolosa* isolates to those from a prior *B. dolosa* outbreak (a) Same tree as in **Fig. 2a**, with isolates from the Lieberman and Michel et al.[15] study expanded and Patient J's isolates compressed. Isolates collected in the prior outbreak are colored by patient. We also included an outgroup isolate X-3-7 (in black). Isolates from Lieberman and Michel et al. are named according to patient and time as previously (ex. C-14-11 was recovered from Patient C, 14 years and 11 months after isolation of the first outbreak strain). Samples with a "-B" at the end of their label indicate a blood source; otherwise, all remaining samples profiled in 2011 were collected from sputum. Isolates with the same subject ID and date are distinguished by letters (ex. J-13-6a-B and J-13-6b). (b) A zoom in on Patient J's samples from Lieberman and Michel et al.'s[15] investigation enables positioning of new samples in context to those obtained a decade prior. Clade names correspond to the tree in **Fig. S2**.

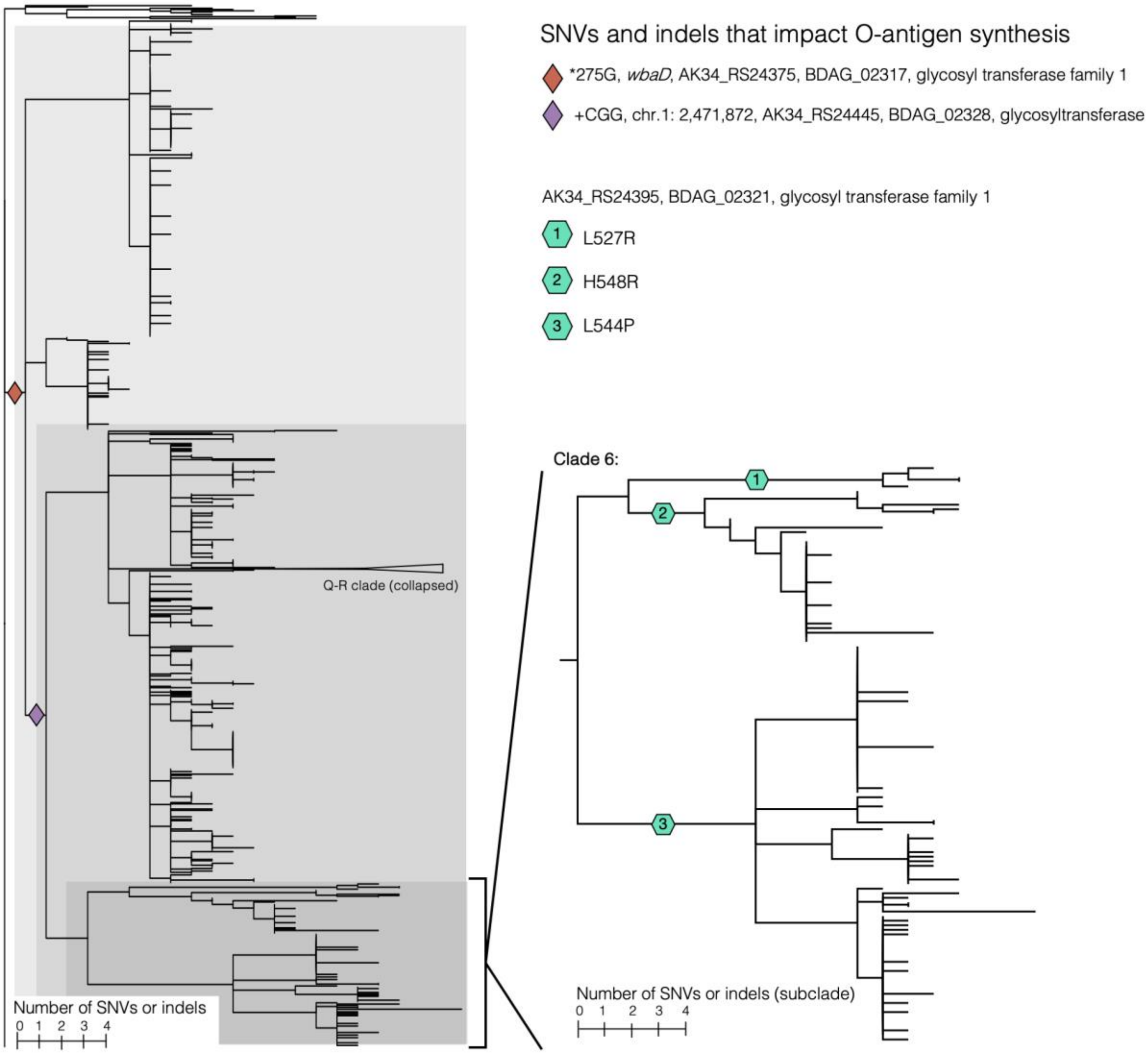

Supp. Figure 4: Over long-term infection, *B. dolosa* accumulates multiple SNVs and indels that impact O-antigen synthesis. A maximum parsimony phylogeny of isolates from Patients J, R, and Q generated from both SNVs and O-antigen-affecting indels, where each indel is represented as a single, independent mutation event (Methods). Diamonds and hexagons demark the emergence of O-antigen-affecting mutations. Clade Q-R is collapsed for simplicity, and a zoom-in on clade 6 reveals clustered O-antigen-affecting mutations. Of 809 strains acquired from Patient J's autopsy, 11 (1.4%, clade 1, white) contain a stop codon, while 317 (39%, clades 2 & 3, light gray) contain only a *wbaD* (BDAG_02317/AK34_RS24375) stop codon reversion. A total of 355 (44%, clades 4, 5, 11 ungrouped samples, and 4 isolates within the Q-R clade, medium gray) contain a further +CGG insertion in BDAG_02328 (AK34_RS24445), and 126 (16%, clade 6, dark gray) contain one of three additional SNVs in BDAG_02321 (AK34_RS24395).

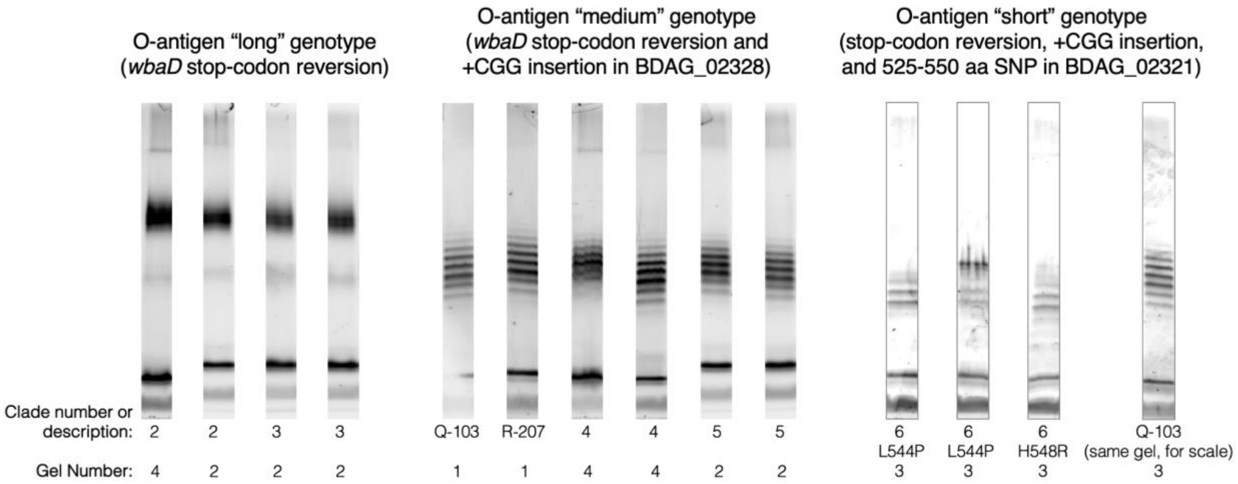

Supp. Figure 5: Different O-antigen phenotypes coexist in *B. dolosa* lung infection. O-antigen phenotypes of diverse *B. dolosa* clones from across **Fig. S4**'s phylogeny. Strains containing only a *wbaD* stop codon reversion (clades 2 and 3) display a "long" banding pattern. Strains containing a +CGG insertion in BDAG_02328 (AK34_RS24445) present a "medium" banding pattern (clades 4,5, Q-R, and 11 ungrouped samples); this is the phenotype that is inferred to have first infected Patients Q and R. Isolates from clade 6 display acquired one of three mutations between amino acids 527 and 548 of BDAG_02321 (AK34_RS24395). Phenotypes of both BDAG_02321 L544P and H548R display a "short" banding pattern; the third independent BDAG_02321 mutation (L527R) was not phenotyped. To contextualize the "short" banding length, a "medium" length isolate (Q-103) imaged on the same gel is shown to the right. Two "standard" isolates, Q-103 and R-221, were imaged on all gels to aid in banding length comparison; raw gel images with these "standard" isolates can be found under the Github repo: ajporet/b_dolosa_evolution. Gel numbers correspond to file names in Github.

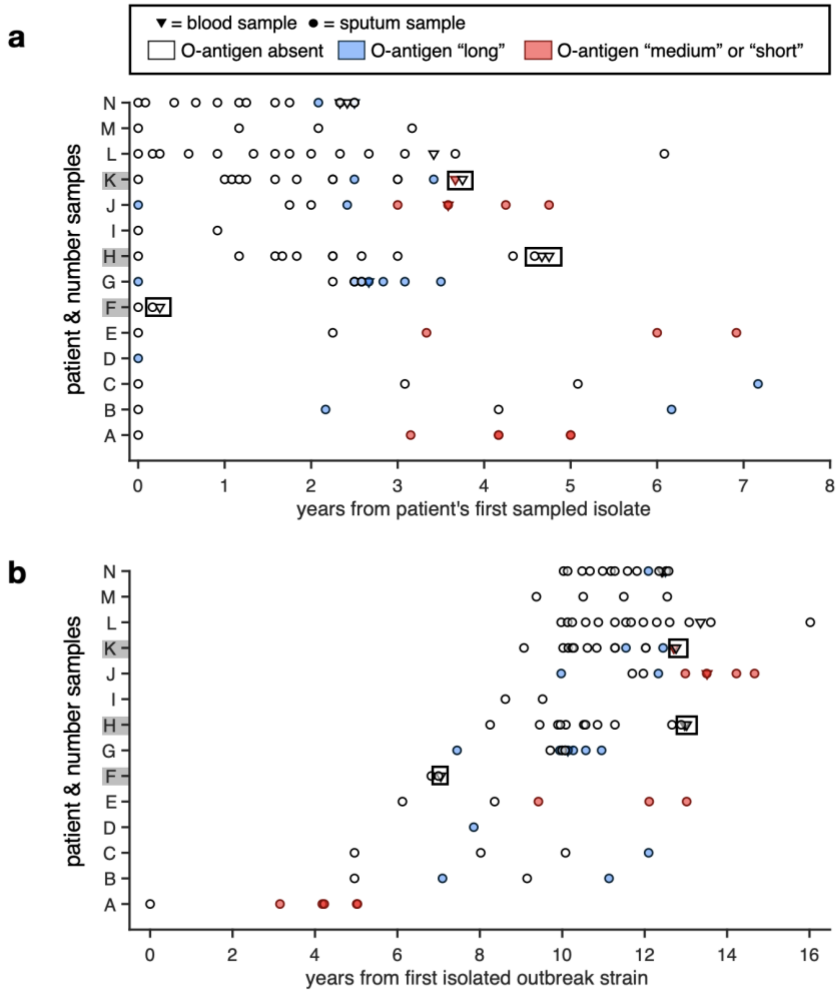

Absent	Long	Medium/ Short	Total Samples
15	2	0	17
4	0	0	4
15	0	0	15
12	2	1	15
2	2	5	9
2	0	0	2
13	0	0	13
3	10	0	13
3	0	0	3
2	0	3	5
0	1	0	1
3	1	0	4
2	2	0	4
1	0	5	6

Supp. Figure 6: Comparing sampling date and time to O-antigen genotype reveals strain coexistence. (a) O-antigen banding patterns predicted for 112 previously-published *B. dolosa* genomes[15] collected during a historical outbreak. Each isolate is represented by a single, shaded dot; the color of each data point represents its predicted O-antigen phenotype. The shape of each datapoint represents the colony’s anatomical origin (triangle = blood, circle = sputum). Isolates that contain a *wbaD* stop codon are classified as “absent " (black). Patients who later developed cepacia syndrome are highlighted in gray in the leftmost margin; isolates taken from the month before and during cepacia syndrome are boxed in black. Those with a stop codon reversion are marked as “long” (blue), and those with an additional mutation in a gene predicted to affect O-antigen function are categorized as “medium or short” (red, **Fig S7**). In 5 out of 14 patients (N, K, J, G, B) ancestral, O-antigen-absent strains are recovered years after an O-antigen-expressing (long or medium/short) strain, highlighting co-existence of phenotypes. A table tabulating the number of isolates with different genotypes is shown to the right. (b) This data is plotted relative to the date when the first outbreak sample was isolated. Of the four blood isolates during cepacia syndrome taken in this previous study, three were O-antigen negative and one was O-antigen medium/short; both O-antigen “long” and “medium/short” strains were found in Patient J’s blood during cepacia syndrome (see **Fig S9**).

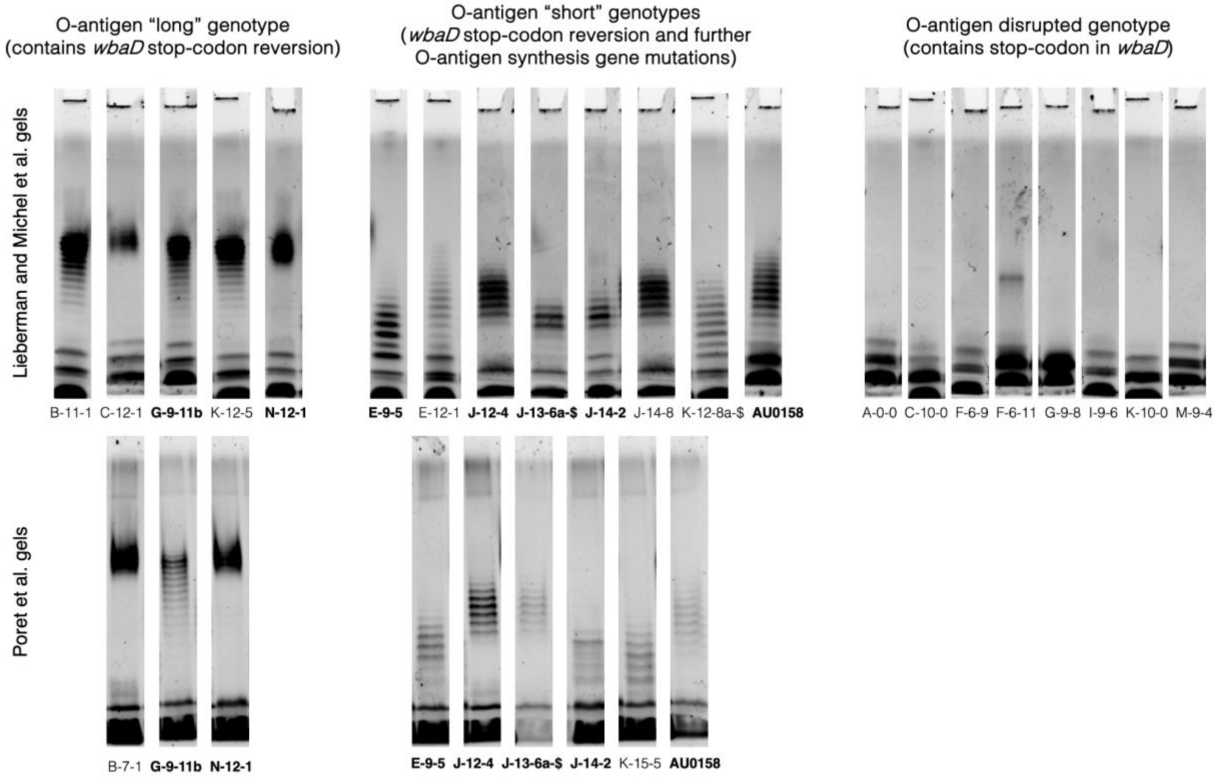

Supp. Figure 7: Reanalysis of a prior *B. dolosa* outbreak reveals O-antigen modification beyond an initial gain-of-function mutation. O-antigen banding patterns of *B. dolosa* isolates from a historical 14-person outbreak. O-antigen phenotypes produced by Lieberman and Michel et al.[15] can be grouped into three classes with an associated genotypic signature. "Disrupted" banding patterns contain a stop codon in gene *wbaD* and "long" banding patterns revert this stop codon. All isolates that acquire a further mutation in an O-antigen-affecting gene display a "short" banding pattern. The exact banding length of "short" isolates varies depending on the O-antigen-affecting mutation and genotypic background. To ensure compatibility between newly created gels and those published by Lieberman and Michel et al.[15], previously-published isolates were re-phenotyped. Bolded sample names indicate an isolate is present in both datasets. Banding patterns appear the same between both analyses.

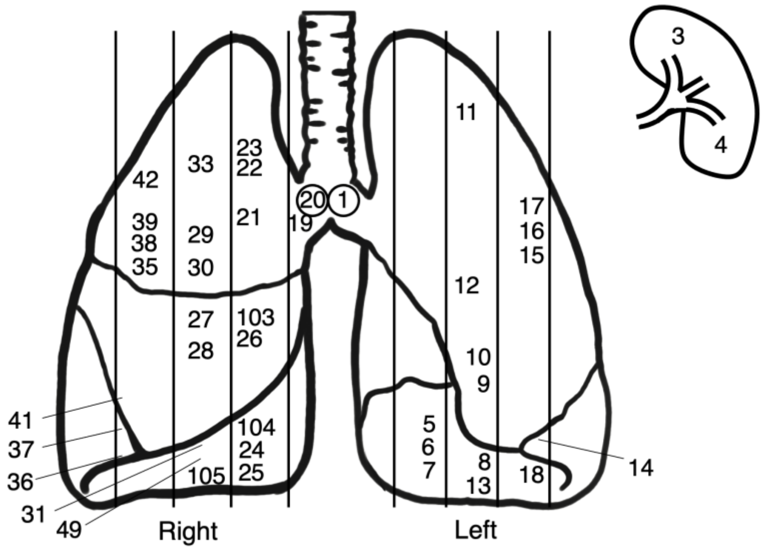

Supp. Figure 8: Diagram of lung sites sampled in Patient J (autopsy). The anatomical location of each sampled lung site is diagrammed. Vertical lines indicate dissection cut sites. Circled numbers indicate that sampled tissue is from a mediastinal lymph node. Samples 3 and 4 were taken from splenic tissue. Lung figure is modified from a diagram by Patrick J. Lynch and C. Carl Jaffe (goo.gl/iC8AjM), CC-BY-2.5.

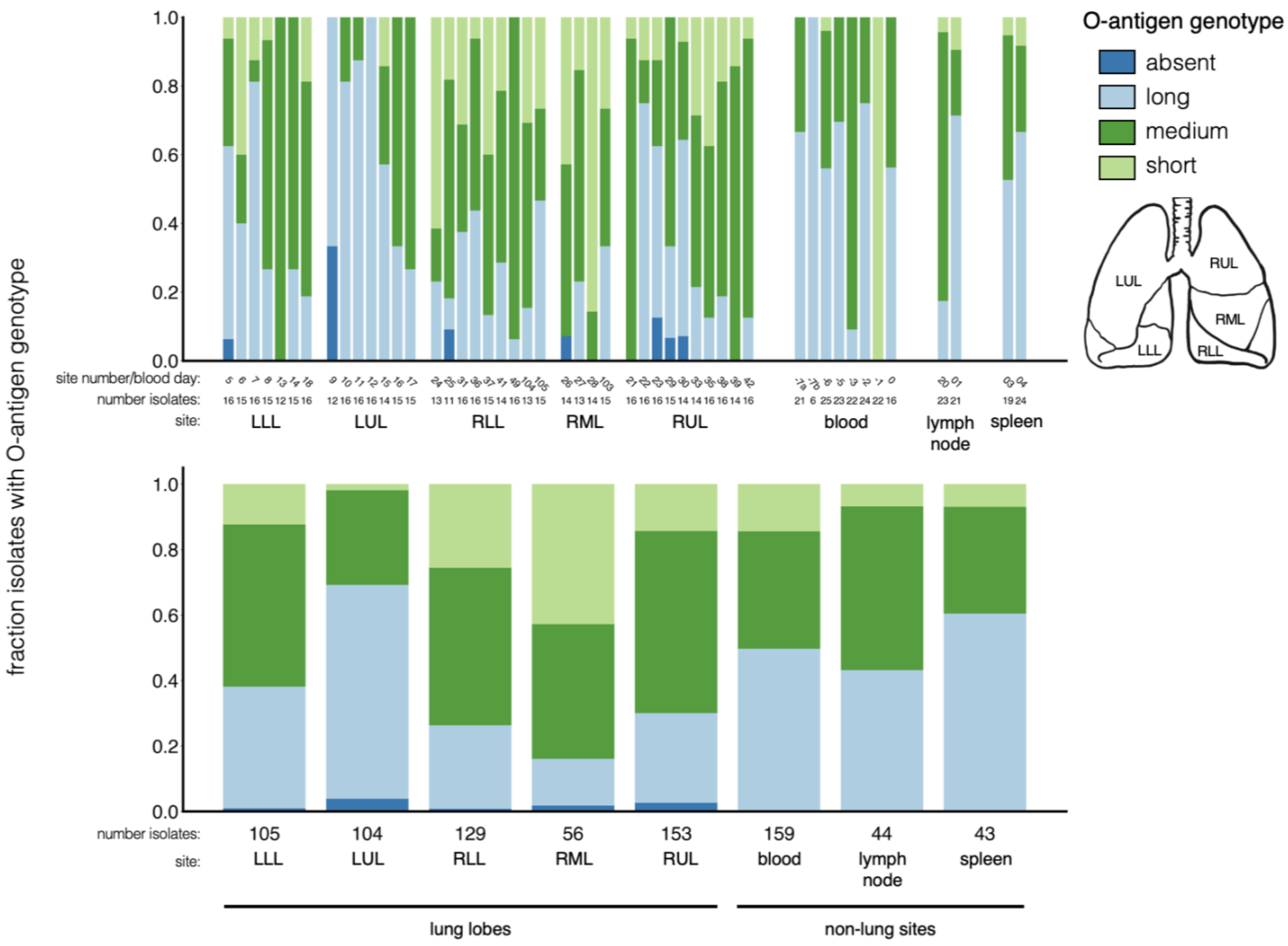

Supp. Figure 9: Distribution of O-antigen genotypes across lung lobes. The fraction of O-antigen genotype classes (as defined in main text and **Fig. S4, S5**) is grouped by anatomical site. Lung, spleen, and lymph node isolates are stratified by sampling site. Blood culture isolates are separated by day of acquisition with 0 marking the day of death. A week prior to death, two independent blood samples were cultured; these are marked as "-7a" and "-7b." Ancestral, O-antigen-absent genotypes are found in all 5 lung lobes. Modest differences in long, medium, and short genotype abundance are observed among lobes ($P < 10^{-10}$; Chi square test, one-tailed). Lung figure is modified from a diagram by Patrick J. Lynch and C. Carl Jaffe (goo.gl/iC8AjM), CC-BY-2.5.

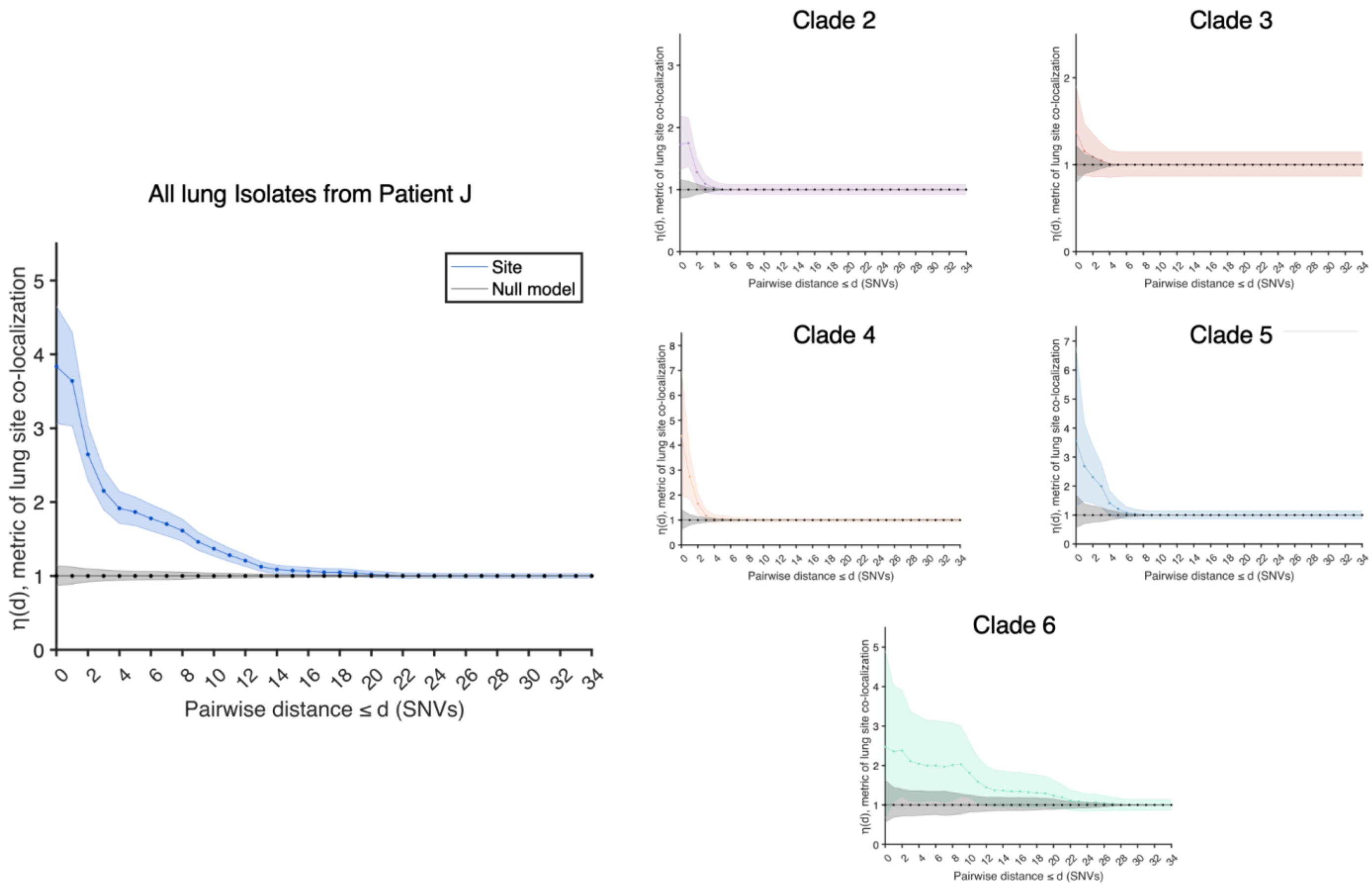

Supp. Figure 10: Weak spatial segregation of *B. dolosa* genotypes is observed within a CF lung autopsy. Using the methods published in Chung et al.[21], we examined whether *B. dolosa* genotypes within Patient J's lung are spatially segregated; i.e, that the same genotype is likely to cluster within the same lung site. The black line indicates the null model corresponding to randomizing sample identity labels within the lung (1,000 trials), and error bars indicate a 95% confidence interval. We calculated the probability of lung-site colocalization for all of Patient J's isolates and each major clade to account for niche evolution that could render one clade spatially diffuse or concentrated. While some spatial correlation is observed, this spatial signal decays after 10 SNVs (~5 years according to previously published molecular clock[15]), suggesting more rapid mixing than has been reported for either *S. maltophilia* or *P. aeruginosa*[21,22].

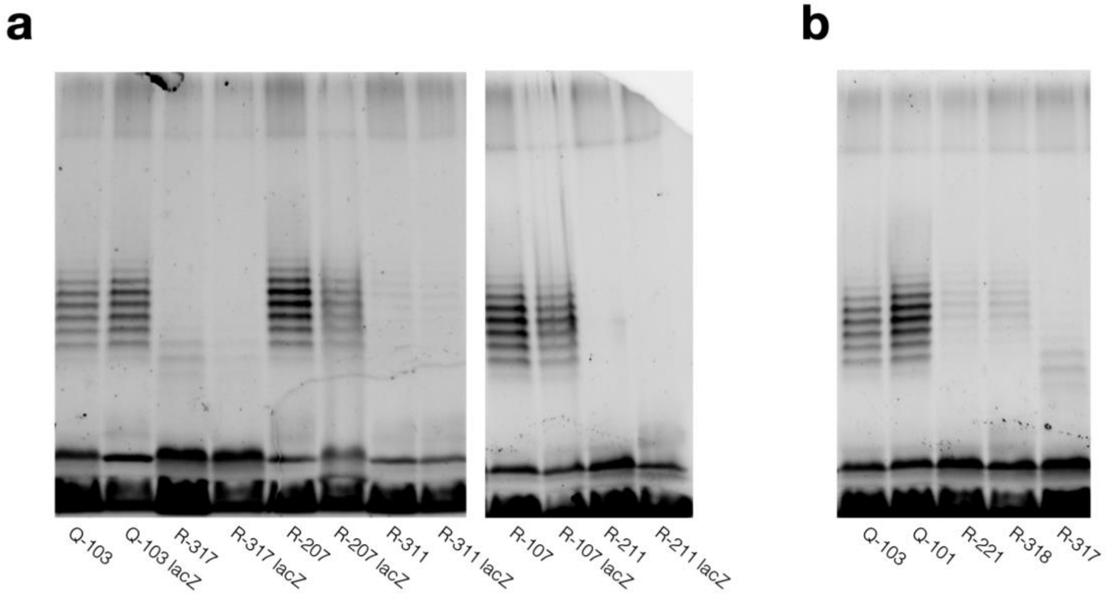

Supp. Figure 11: Lipopolysaccharide (LPS) staining shows O-antigen differences between strains used in murine model and macrophage assays. (a) Each pair of *B. dolosa* strains used in the murine infection model were extracted and imaged on the same gel. O-antigen staining patterns show no obvious differences between lacZ marked and unmarked strains. (b) All strains pairs in the **Fig. S13b** macrophage invasion experiments were imaged on the same gel, displaying the expected O-antigen phenotype.

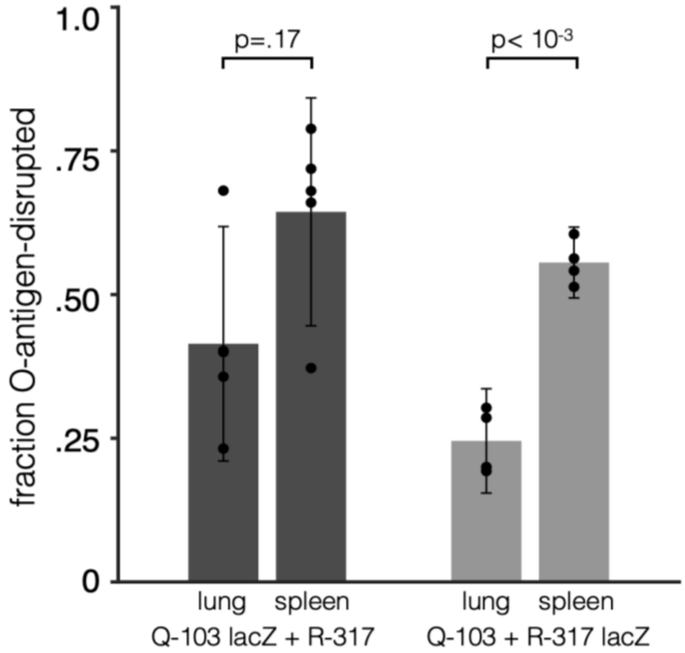

Supp. Figure 12: LacZ cassette insertion into *B. dolosa* does not impact a murine infection model outcome. To ensure the LacZ cassette insertion used to differentiate between O-antigen mutant and wild type strains did not bias strain fitness, we conducted a label swap in which a LacZ cassette was integrated into both Q-103 and R-317 strains separately. Experiments were performed with both labeled pairs on the same day. Competitions of Q-103 lacZ vs. R-317 and Q-103 vs. R-317 lacZ showed similar results to those in **Fig. 4a** (two-sided paired T-test, Q-103 lacZ vs. R-317: P= .17, Q-103 vs. R-317 lacZ: $P < 10^{-3}$). Outlier points seen in the Q-103 lacZ vs. R-317 lung and spleen occur within the same mouse. Data presented here represent a replicate from that shown in **Fig. 4a**; results in **Fig. 4a** come from 2 experimental batches containing all 3 strain pairs shown.

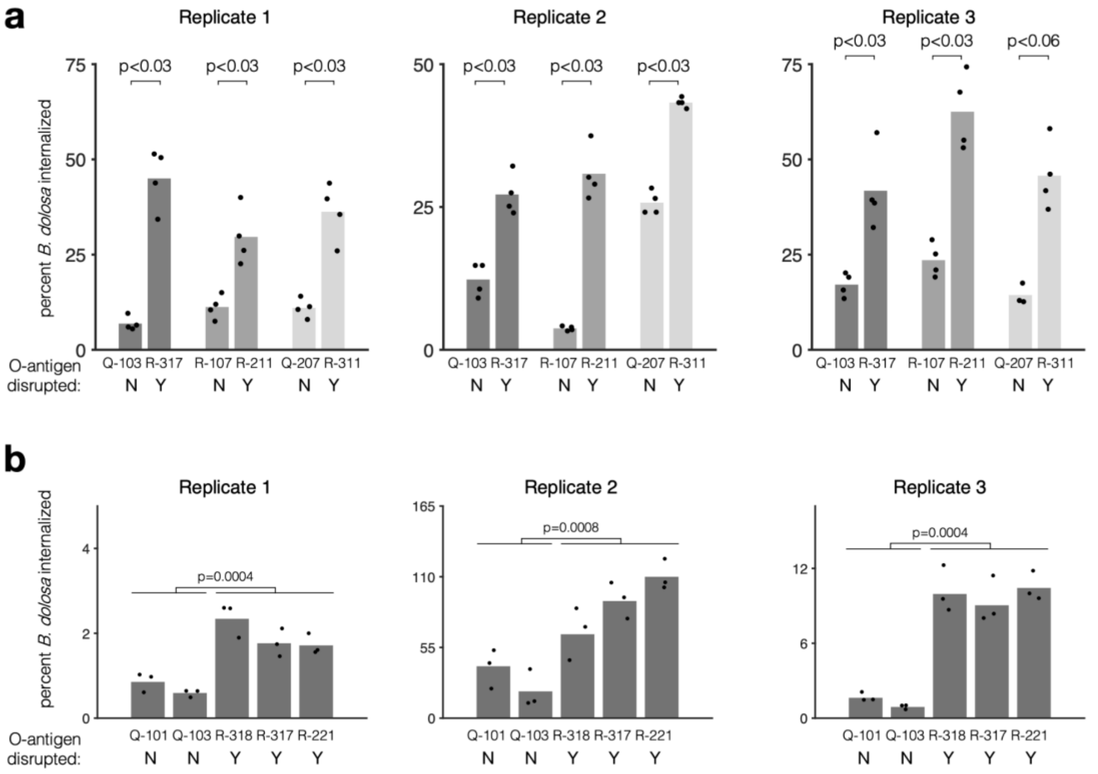

Supp. Figure 13: Kanamycin exclusion assays demonstrate that O-antigen disruption increases *B. dolosa* internalization/survival within macrophages. Macrophages were infected for 2 hours with *B. dolosa* strains with and without an O-antigen-disrupting mutation and then incubated for 2 hours with kanamycin, which kills extracellular bacteria. Pairs of near-isogenic strains are shown adjacent to one another. The number of intracellular bacteria is normalized by the number of bacteria obtained from an identical culture without kanamycin treatment. (a) All *B. dolosa* strain pairs used in the murine experiments were compared against each other, demonstrating significant differences in macrophage invasion ($P < .06$, two-sided Wilcoxon rank sum test, p-values are uncorrected). Replicate 1 is also shown in **Fig. 4b**. (b) A separate panel including two O-antigen wild-type (Q-103, Q-101) and mutant (R-318, R-317, R-221) strains display the same expected phenotype as shown in **Fig. 4b**'s macrophage assays. All measurements of O-antigen mutant strains are aggregated and compared to O-antigen wild-type strains ($P < .001$, two-sided Wilcoxon rank sum test, p-values are uncorrected).

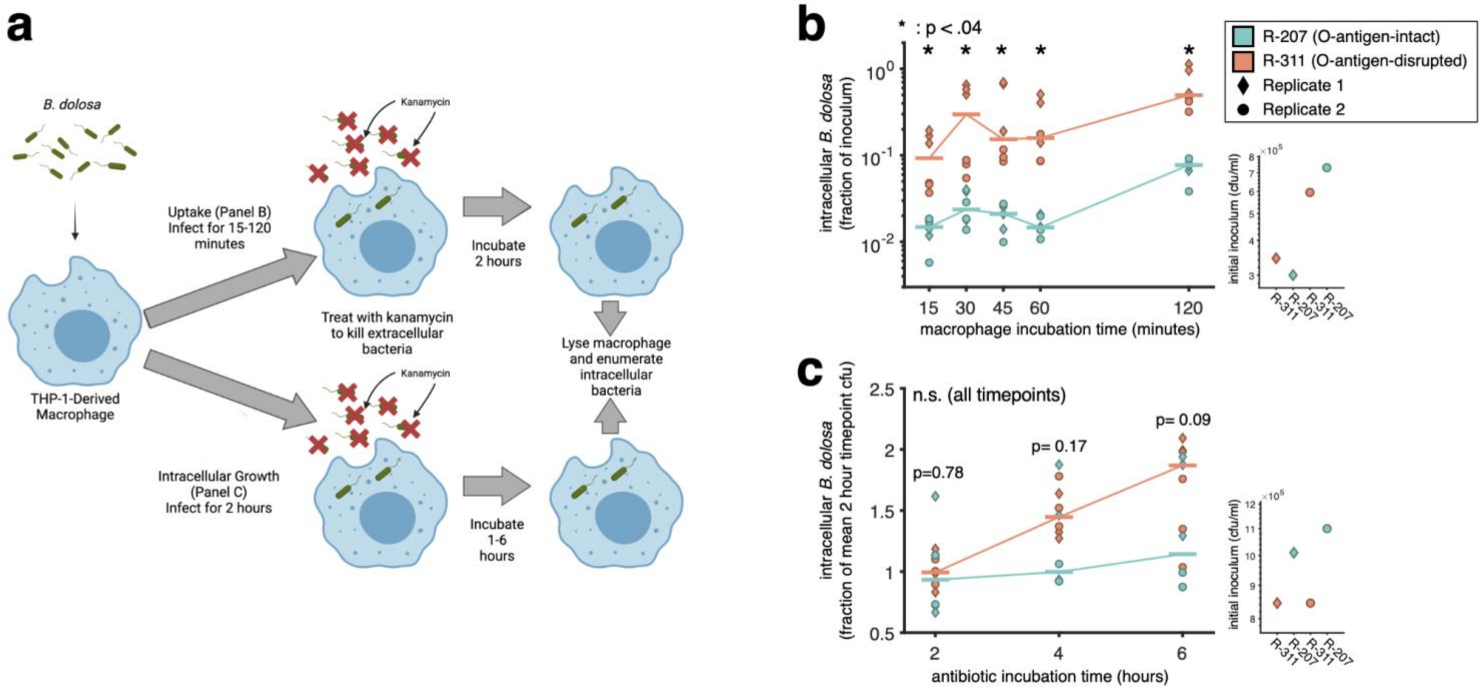

Supp. Figure 14: O-antigen disruption in *B. dolosa* causes more rapid internalization by macrophage-like cells. (a) Macrophage-like cells were infected with *B. dolosa* for varying durations (b) and incubated with antibiotics for varying times (c) to assess the impact of O-antigen disruption on uptake and intracellular growth, respectively. Horizontal lines represent medians in both figures. Two trials of each experiment were run with near-isogenic *B. dolosa* strains R-207 (intact O-antigen) and R-311 (disrupted O-antigen). We find the disrupted O-antigen strain R-311 displays increased internalization, but not prolonged survival or increased growth rate in macrophages. When the macrophage incubation time is varied (b), the O-antigen-disrupted R-311 strain showed consistently higher intracellular recovery than the O-antigen-intact R-207 strain across all timepoints (two-sided Wilcoxon rank-sum test $p < 0.04$ for all time points, replicates combined across trials). In contrast, when both *B. dolosa* strains are incubated with antibiotics for varying time points following initial internalization (c), we see no significant difference in bacterial growth rate between strains (two-sided Wilcoxon rank-sum test $p > .05$ for all time points, replicates combined across trials). Intrallecular *B. dolosa* CFU at each time point is normalized to the mean CFU at two hours for that strain in that trial. This indicates that the disrupted O-antigen strain does not exhibit an increased replication rate within macrophages. For both experiments, the initial inoculum cfu for each trial is shown on the right. (a) was created in BioRender. Schaefers, M. (2025) https://BioRender.com/q34e053.

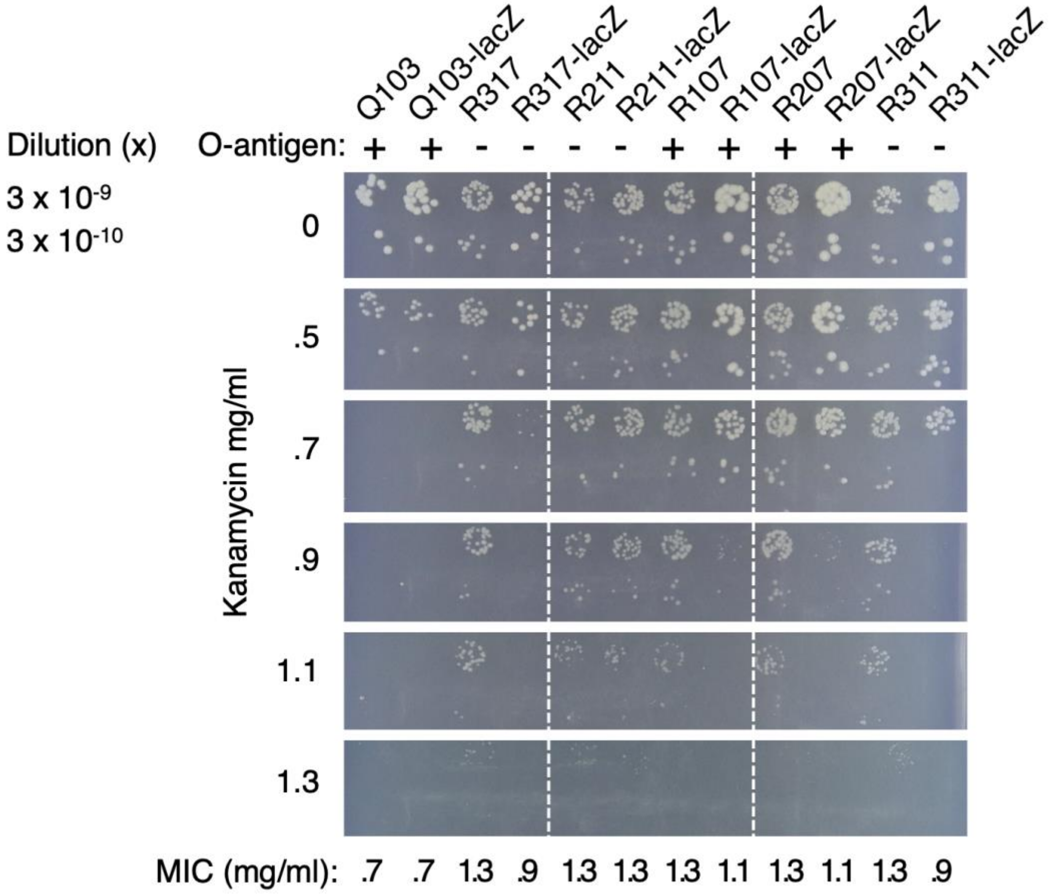

Figure S15: Near-isogenic *B. dolosa* O-antigen disrupted and intact pairs show little difference in kanamycin minimum inhibitory concentration (MIC) To assess differences in kanamycin resistance between O-antigen intact and disrupted *B. dolosa* strains, we measured the minimum inhibitory concentration (MIC) for strains R-317, Q-103, R-211, R-107, R-311, and R-207, along with their lacZ-modified counterparts. Near-isogenic strain pairs are separated by dotted lines. Following overnight growth to saturation, 3 µL of diluted cultures (10^{-2} and 10^{-4} - 10^{-10} 10-fold serial dilutions) were spot plated onto media containing varying kanamycin concentrations (0, 0.5, 0.7, 0.9, 1.1, and 1.3 mg/mL).Following incubation, colony counts were obtained from the 10^{-9} (top row) and 10^{-10} (bottom row) total dilutions. MIC was defined as the highest kanamycin concentration with no visible colonies across both dilutions. The lacZ insertion appears to influence kanamycin resistance, likely due to a gentamicin resistance marker introduced during cloning. While the pair R-317 and Q-103 exhibited slight differences-- the O-antigen intact strain R-317 showed an MIC of 0.7 mg/mL, whereas Q-103, also O-antigen intact, had an MIC of 0.9 mg/mL -- these MICs remain below the 1 mg/mL threshold used in kanamycin resistance assays to evaluate *B. dolosa* phagocytosis and survival in macrophages. Pairs R-211/R-107 and R-311/R-207 displayed identical resistance profiles.
