# Supplementary material for: De novo mutations mediate phenotypic switching in an opportunistic human lung pathogen
Source: Nat Commun. 2025 Jul 23;16:6799. doi: 10.1038/s41467-025-61168-4 (PMC12287342; doi:10.1038/s41467-025-61168-4)
Supplement: Supplementary file 2 — Description of Additional Supplementary Files [file 41467_2025_61168_MOESM2_ESM.docx]

**Description of Additional Supplementary Files**

File Name: Supplementary Data 1

Description: **Sources of B. dolosa isolates.** Metadata describing each anatomical sample used in this study. Tissue condition and cut number are described in the “Gross description from autopsy” and “Lung cut” columns. “Timing of isolation" describes when a sample was acquired. For Patient J, time point 0 corresponds to the day of autopsy, and negative numbers indicate days prior to death. For patients Q and R, numbers indicate the months from first detected infection (which occurred approximately at the same time as Patient J's autopsy). "Sequencing labels (used in Github)", describes how samples are labeled in our Github Repo. Patient J is referred to as P02, Patient Q as P06, and Patient R as P07. LT corresponds to lung tissue, LG to lymph node, and SL spleen. Samples are formatted as: "Patient Tissue Type - Lung Site XX", where XX indicates the B. dolosa isolate number. For example, "P02LT-4203" indicates Patient 2/J, lung site 42, isolate 3. "P06SPv-311" indicates Patient 6/Q, time point +38 months, isolate 11. Some samples are labeled without a dash when the site description consists of 3 or more characters.

File Name: Supplementary Data 2

Description: **O-antigen-related genes mutated during a miniature outbreak of B. dolosa.** O-antigen-related gene mutations observed during a localized outbreak of B. dolosa in Patients J, Q, and R. This table consolidates all insertions, deletions, and single nucleotide variants (SNVs) predicted to affect O-antigen synthesis and expression. Rows highlighted in grey indicate de novo mutations that emerged independently within Patients Q or R.

File Name: Supplementary Data 3

Description: **Reanalysis of mutations affecting the O-antigen in isolates collected 5–11 years prior to autopsy in Patient J.** Each column represents one isolate, and each row corresponds to a mutation. A "1" indicates the presence of a mutation in a sample, while blanks denote a wild-type genotype. Notably, all isolates carry the C611R mutation in BDAG_02321 (AK34_RS24395) relative to the AU0158 reference. Phylogenetic reconstruction suggests that this C611R mutation emerged uniquely within the AU0518 strain and is absent in other outbreak sequences. All four distinct O-antigen genotypes observed in autopsy isolates were identified in these earlier samples. J_11_8 contains a stop codon in wbaD. J_9_11 reverts the wbaD stop codon and contains no other mutations that impact O-antigen expression. J_12_7, J_12_11, J_13_6b, and J_14_2 contain a further 3 base pair insertion in BDAG_02328 (AK34_RS24445); the blood isolate J_13_6a_B contains this 3 base pair insertion and an additional SNV in BDAG_02321 (AK34_RS24395).

File Name: Supplementary Data 4

Description: **Polymorphic loci mutated during a miniature outbreak of B. dolosa within Patients J, Q, and R.** N's indicate a nucleotide call could not be made at a listed position (see Methods). Samples are named with the format "Patient-Sample Type-Location number (first two numbers) isolate number (second two numbers). LT = lung tissue, BL = blood, SL = spleen, and LG = lymph node. For example, sample J-LT-0601 is from lung tissue site 6, isolate 1 from Patient J.

In gene descriptions, NAs indicate that a matching gene annotation could not be found (for example, no matching BDAG annotation could be found for gene AK34_RS15255, which is now considered a pseudogene in modern protein-annotating programs). If a gene is intergenic, two genes are listed with either a "---" (annotation column) or "-" (all other columns) inbetween.

File Name: Supplementary Data 5

Description: **Genes mutated during a miniature outbreak of B. dolosa within Patient Q & R. NAs** indicate that a matching gene annotation could not be found (for example, no matching BDAG annotation could be found for gene AK34_RS15255, which is now considered a pseudogene in modern protein-annotating programs). If a gene is intergenic, two genes are listed with either a "---" (annotation column) or "-" (all other columns) inbetween. The "Mutation" column indicates the mutations that occurred in this gene. For example, 'K30T' signifies a lysine to threonine mutation at the 30th amino acid residue. A '*' indicates a stop codon.

File Name: Supplementary Data 6

Description: **Genes mutated during a miniature outbreak of B. dolosa within Subject J.** NAs indicate that a matching gene annotation could not be found (for example, no matching BDAG annotation could be found for gene AK34_RS15255, which is now considered a pseudogene in modern protein-annotating programs). If a gene is intergenic, two genes are listed with either a "---" (annotation column) or "-" (all other columns) inbetween. The "Mutation" column indicates the mutations that occurred in this gene. For example, 'K30T' signifies a lysine to threonine mutation at the 30th amino acid residue. A '*' indicates a stop codon.

File Name: Supplementary Data 7

Description: **Insertions and deletions (indels) in and nearby genes affecting the O-antigen presentation.** Indels suspected to impact O-antigen expression are shown for newly-collected isolates from subjects J, Q, and R. Each column describes one isolate, and each row a mutation. A 1 indicates a mutation is present in a sample, a 'Δ' indicates that this row's mutation is fully contained within a region deleted within that specific isolate, and a "?" indicates that coverage at that position was too low for a definitive call. The fraction of all J, Q, and R's isolates containing a mutation, an unclear call, or a deletion are tabulated. Filtering cutoffs and methodology for identifying O-antigen-affecting indels is described in the Methods description 'Mutation detection and phylogenetic inference'.

File Name: Supplementary Data 8

Description: **Unprocessed Breseq output profiling B. dolosa isolates from Patients J, Q, and R.** Samples are named with the format ""Patient-Sample Type-Location number (first two numbers) isolate number (second two numbers). LT = lung tissue, BL = blood, SL = spleen, and LG = lymph node. Each column describes one isolate, and each row a mutation. A 1 indicates a mutation is present in a sample, a 'Δ' indicates that this row's mutation occurred within a region deleted in that specific isolate, and a "?" indicates that coverage at that position was too low for a definitive call.

File Name: Supplementary Data 9

Description: **KEGG Enrichment Analysis**. The number of B. dolosa mutations that fall within different KEGG ontology pathways (B. dolosa T number: T03793) are tabulated for both Subject J and Subjects Q & R combined. P-values are calculated by estimating the probability across 10000 trials that the same or greater number of mutations would randomly occur within a pathway. Significantly enriched pathways are highlighted.

File Name: Supplementary Data 10

Description: **Similarity matrix of B. dolosa KEGG pathways significantly enriched in patients Q and R.** To identify whether all significantly enriched B. dolosa KEGG pathways in patients Q and R are due to a shared set of genes, we calculate the number of genes in common between two pathways/the total genes in each row’s pathway. The pathways “Fructose and mannose metabolism” (00051), “Amino sugar and nucleotide sugar metabolism” (00520), and “O-Antigen nucleotide sugar biosynthesis” (00541) all share at least 25% of the same genes, suggesting a single signal may be driving driving enrichment significance among all these pathways .

File Name: Supplementary Data 11

Description: **SNVs identified as potential recombination or multi-nucleotide mutation events.** To investigate potential recombination or multi-nucleotide mutation events that could confound phylogenetic interpretations, we screened for pairs of closely located SNVs (<1,000 bp apart) that were perfectly correlated in their presence across the phylogeny. This analysis revealed five small multi-variant blocks, ranging in size from 1 to 15 base pairs (bp), encompassing a total of 11 SNPs. The table provides details on the nucleotide position, mutation, gene name, annotation, and samples affected by each SNV. Horizontal lines separate the recombination blocks for clarity.

File Name: Supplementary Data 12

Description: **Unprocessed Breseq output profiling isolates from prior outbreak of B. dolosa.** Isolates are labeled according to patient and time (ex. C-14-11 was recovered from Patient C, 14 years and 11 months after isolation of the first strain). 2011 samples with a “-B” at the end of their label indicate a blood source; otherwise, all remaining 2011 samples were collected from sputum. Each column describes one isolate, and each row a mutation. To increase detection sensitivity, Breseq was run in "predict-polymorphisms" mode, which outputs the percentage of reads that match a predicted mutation. Alternatively,a 'Δ' indicates that this row's mutation is fully contained within a region deleted within that specific isolate, and a "?" indicates that coverage at that position was too low for a definitive call. Notably, samples G_10_2, H_10_10,J_11_11, K_11_3b, N_10_0, J_12_4, J_14_8, E_13_0, L_10_10, L_12_7 are not shown in this analysis due to incomplete processing of one or more chromosomes.
