# Supplementary material for: De novo mutations mediate phenotypic switching in an opportunistic human lung pathogen
Source: Nat Commun. 2025 Jul 23;16:6799. doi: 10.1038/s41467-025-61168-4 (PMC12287342; doi:10.1038/s41467-025-61168-4)
Supplement: Supplementary file 4 — Reporting Summary [file 41467_2025_61168_MOESM4_ESM.pdf]

nature portfolio

Corresponding author(s): Tami Lieberman, Gregory Priebe

Last updated by author(s): Apr 17, 2025

Reporting Summary

Nature Portfolio wishes to improve the reproducibility of the work that we publish. This form provides structure for consistency and transparency in reporting. For further information on Nature Portfolio policies, see our Editorial Policies and the Editorial Policy Checklist.

Statistics

For all statistical analyses, confirm that the following items are present in the figure legend, table legend, main text, or Methods section.

n/a	Confirmed	
☐	☒	The exact sample size (*n*) for each experimental group/condition, given as a discrete number and unit of measurement
☐	☒	A statement on whether measurements were taken from distinct samples or whether the same sample was measured repeatedly
☐	☒	The statistical test(s) used AND whether they are one- or two-sided<br>*Only common tests should be described solely by name; describe more complex techniques in the Methods section.*
☐	☒	A description of all covariates tested
☐	☒	A description of any assumptions or corrections, such as tests of normality and adjustment for multiple comparisons
☐	☒	A full description of the statistical parameters including central tendency (e.g. means) or other basic estimates (e.g. regression coefficient) AND variation (e.g. standard deviation) or associated estimates of uncertainty (e.g. confidence intervals)
☐	☒	For null hypothesis testing, the test statistic (e.g. *F*, *t*, *r*) with confidence intervals, effect sizes, degrees of freedom and *P* value noted<br>*Give P values as exact values whenever suitable.*
☒	☐	For Bayesian analysis, information on the choice of priors and Markov chain Monte Carlo settings
☒	☐	For hierarchical and complex designs, identification of the appropriate level for tests and full reporting of outcomes
☒	☐	Estimates of effect sizes (e.g. Cohen's *d*, Pearson's *r*), indicating how they were calculated

Our web collection on statistics for biologists contains articles on many of the points above.

Software and code

Policy information about availability of computer code

Data collection	All code used in this project to collect and process sequence data is published under https://github.com/ajporet/b_dolosa_evolution.
Data analysis	All custom scripts and code used in this project are published under https://github.com/ajporet/b_dolosa_evolution. These scripts utilize publicly available packages and programs: cutadapt v1.18, sickle v1.33, bowtie2, SAMtools v1.5, Phylip v3.69, Breseq v0.30.0.

For manuscripts utilizing custom algorithms or software that are central to the research but not yet described in published literature, software must be made available to editors and reviewers. We strongly encourage code deposition in a community repository (e.g. GitHub). See the Nature Portfolio guidelines for submitting code & software for further information.

Data

Policy information about availability of data

All manuscripts must include a data availability statement. This statement should provide the following information, where applicable:

- Accession codes, unique identifiers, or web links for publicly available datasets
- A description of any restrictions on data availability
- For clinical datasets or third party data, please ensure that the statement adheres to our policy

Sequences from B. dolosa isolates obtained in this study are uploaded to NCBI under BioProject PRJNA1063312.

Research involving human participants, their data, or biological material

Policy information about studies with human participants or human data. See also policy information about sex, gender (identity/presentation), and sexual orientation and race, ethnicity and racism.

Reporting on sex and gender	Sex and gender are not reported for study patients due to privacy and identifiability concerns.
Reporting on race, ethnicity, or other socially relevant groupings	Race and ethnicity are not reported for study patients due to privacy and identifiability concerns.
Population characteristics	3 adult patients with CF were recruited as part of the new infection cluster. The index patient (Patient J) was age 30-40 years, and Patients Q and R were age 20-30 years
Recruitment	The patients were identified by study investigators including CF clinicians (AZU) as well as the director of the clinical microbiology lab (AJM) who was made aware of any new patients growing B. dolosa. Due to the known high transmissibility of B. dolosa, it was standard practice to refer any patient growing B. dolosa to BCH. Thus, it is very unlikely that any additional patients related to this new cluster were missed.
Ethics oversight	The study was approved by the Boston Children's Hospital IRB (protocol 05-02-014R), and informed consent was obtained for sample use/collection and medical record review.

Note that full information on the approval of the study protocol must also be provided in the manuscript.

Field-specific reporting

Please select the one below that is the best fit for your research. If you are not sure, read the appropriate sections before making your selection.

☒ Life sciences ☐ Behavioural & social sciences ☐ Ecological, evolutionary & environmental sciences

For a reference copy of the document with all sections, see nature.com/documents/nr-reporting-summary-flat.pdf

Life sciences study design

All studies must disclose on these points even when the disclosure is negative.

Sample size	For B. dolosa isolate collection, we chose to sample 24 isolates per sputum sample and autopsy site based on prior research demonstrating this depth as mostly representative of diversity within a single sample (Lieberman et al. 2014, 10.1038/ng.2848). To evaluate the diversity of O-antigen phenotypes, we selected representative isolates aiming to sample each major clade at least once within the phylogenies of Patient J's lung autopsy and Patients Q and R's sputum. Sample sizes for the murine pneumonia model were determined through a power analysis (Cohen's D = 1.8, alpha=.05) using a predicted effect size derived from a similar murine model that competed B. dolosa strains differing by a gene also identified as under selection during infection (Schaefers et al. 2021, 10.1128/mbio.01823-21). For kanamycin exclusion assays, a small pilot study was conducted with n=3 replicates due to equipment and personnel constraints. Subsequent data analysis revealed significant results, leading to the decision not to augment the sample size further.
Data exclusions	Filtering steps used to exclude poor quality sequencing data is described in the Methods section "Mutation detection and phylogenetic inference". Data was excluded from the murine pneumonia model if accurate counts of B. dolosa isolates could not be determined from a petri plate due to colony overcrowding. No data was excluded from the kanamycin exclusion assays.
Replication	All replication attempts of kanamycin exclusion assays and murine competition experiments were successful.
Randomization	This study chronicles the evolution of clinically derived bacteria and identifies differences between naturally occurring strains. This study therefore contains no treatment groups or experimental intervention that would require randomization.
Blinding	This study contains no experimental intervention or treatment groups that would require researcher blinding.

Reporting for specific materials, systems and methods

We require information from authors about some types of materials, experimental systems and methods used in many studies. Here, indicate whether each material, system or method listed is relevant to your study. If you are not sure if a list item applies to your research, read the appropriate section before selecting a response.

Materials & experimental systems

n/a	Involved in the study
☒	☐ Antibodies
☐	☒ Eukaryotic cell lines
☒	☐ Palaeontology and archaeology
☐	☒ Animals and other organisms
☐	☒ Clinical data
☒	☐ Dual use research of concern
☒	☐ Plants

Methods

n/a	Involved in the study
☒	☐ ChIP-seq
☒	☐ Flow cytometry
☒	☐ MRI-based neuroimaging

Eukaryotic cell lines

Policy information about cell lines and Sex and Gender in Research

Cell line source(s)	Human THP-1 monocytes (ATCC)
Authentication	Human THP-1 monocytes were not authenticated.
Mycoplasma contamination	Human THP-1 monocytes were not tested for mycoplasma contamination.
Commonly misidentified lines (See ICLAC register)	No commonly misidentified cell lines were used.

Animals and other research organisms

Policy information about studies involving animals; ARRIVE guidelines recommended for reporting animal research, and Sex and Gender in Research

Laboratory animals	C57BL/6 mice, female, 6 to 8 weeks of age (Taconic Biosciences)
Wild animals	This study did not involve wild animals.
Reporting on sex	This study's murine pneumonia model utilizes exclusively female mice. Female mice exhibit less less size variation at 6-8-week olds old compared to male mice, which results in lower mouse-to-mouse variability in the amount of bacteria translocated to the lungs from the nose after intranasal inoculation.
Field-collected samples	This study did not involve field-collected samples.
Ethics oversight	All animal experiments were approved by the Boston Children's Hospital Institutional Animal Care and Use Committee under assurance number A3303-01 and protocol number 1241.

Note that full information on the approval of the study protocol must also be provided in the manuscript.

Clinical data

Policy information about clinical studies

All manuscripts should comply with the ICMJE guidelines for publication of clinical research and a completed CONSORT checklist must be included with all submissions.

Clinical trial registration	N/A
Study protocol	The study (not a clinical trial) was approved by the Boston Children's Hospital IRB (protocol 05-02-014R), and informed consent was obtained for sample use/collection and medical record review.
Data collection	A description of sample collection protocols is included under the methods subsection: "Study cohort, isolate sampling, and genome sequencing."
Outcomes	N/A

Plants

Seed stocks	N/A
Novel plant genotypes	N/A
Authentication	N/A
