# Supplementary material for: De novo mutations mediate phenotypic switching in an opportunistic human lung pathogen
Source: Nat Commun. 2025 Jul 23;16:6799. doi: 10.1038/s41467-025-61168-4 (PMC12287342; doi:10.1038/s41467-025-61168-4)
Supplement: Supplementary file 5 — Transparent Peer Review file [file 41467_2025_61168_MOESM5_ESM.pdf]

nature portfolio

Peer Review File

De novo mutations mediate phenotypic switching in an opportunistic human lung pathogen

Corresponding Author: Professor Tami Lieberman

This manuscript has been previously reviewed at another journal. This document only contains information relating to versions considered at Nature Communications.

This file contains all reviewer reports in order by version, followed by all author rebuttals in order by version.

Version 0:

Reviewer comments:

Reviewer #1

(Remarks to the Author)
I thank the reviewers for comprehensively dealing with all of my concerns and errors that I noted on a previous version of this manuscript. I am satisfied that it is now acceptable for publication. The work will be of broad interest and I think they have demonstrated clear novelty

(Remarks on code availability)
I reviewed the code in the previous revision, but not for this revision as no substantial changes to analysis pipelines was carried out that I know of

Reviewer #2

(Remarks to the Author)
The authors have nicely addressed all my comments

(Remarks on code availability)

Reviewer #3

(Remarks to the Author)
I thank the authors for addressing my critiques to the best of their ability, given the lack of additional data/samples from enrolled patients.

(Remarks on code availability)

Open Access This Peer Review File is licensed under a Creative Commons Attribution 4.0 International License, which permits use, sharing, adaptation, distribution and reproduction in any medium or format, as long as you give appropriate credit to the original author(s) and the source, provide a link to the Creative Commons license, and indicate if changes were made.
In cases where reviewers are anonymous, credit should be given to 'Anonymous Referee' and the source.
The images or other third party material in this Peer Review File are included in the article’s Creative Commons license, unless indicated otherwise in a credit line to the material. If material is not included in the article’s Creative Commons license and your intended use is not permitted by statutory regulation or exceeds the permitted use, you will need to obtain permission directly from the copyright holder.
To view a copy of this license, visit https://creativecommons.org/licenses/by/4.0/
